# Evaluation of Toxicity and Biodegradability of Cholinium Amino Acids Ionic Liquids

**DOI:** 10.1371/journal.pone.0059145

**Published:** 2013-03-15

**Authors:** Xue-Dan Hou, Qiu-Ping Liu, Thomas J. Smith, Ning Li, Min-Hua Zong

**Affiliations:** 1 State Key Laboratory of Pulp and Paper Engineering, College of Light Industry and Food Sciences, South China University of Technology, Guangzhou, China; 2 College of Bioscience and Bioengineering, South China University of Technology, Guangzhou, China; 3 Biomedical Research Centre, Sheffield Hallam University, Sheffield, United Kingdom; RMIT University, Australia

## Abstract

Cholinium amino acid ionic liquids ([Ch][AA] ILs), which are wholly composed of renewable biomaterials, have recently been demonstrated to have very promising properties for applications in organic synthesis and biomass pretreatment. In this work, the toxicity of these ILs toward enzymes and bacteria was assessed, and the effect of the anion on these properties is discussed. The inhibitory potentials of this type of ILs to acetylcholinesterase were weaker approximately an order of magnitude than the traditional IL 1-butyl-3-methylimidazolium tetrafluoroborate. Additionally, the [Ch][AA] ILs displayed low toxicity toward the bacteria tested. Furthermore, the biodegradability of the [Ch][AA] ILs was evaluated via the closed bottle and CO_2_ headspace tests using wastewater microorganisms. All the ILs were classified as ‘readily biodegradable’ based on their high levels of mineralization (62-87%). The presence of extra carboxyl or amide groups on the amino acid side chain rendered the ILs significantly more susceptible to microbial breakdown. In addition, for most of the [Ch][AA] ILs, low toxicity correlated with good biodegradability. The low toxicity and high biodegradability of these novel [Ch][AA] make them promising candidates for use as environmentally friendly solvents in large-scale applications.

## Introduction

In the last two decades, ionic liquids (ILs) have attracted growing interest in various areas such as organic synthesis, catalysis, biocatalysis and biomass pretreatment, owing to their excellent thermal and chemical stability, their outstanding ability to dissolve a broad range of compounds and the fact that their properties can be tuned by individual engineering of the anion and cation components [1], [2]. They are widely regarded as ‘green’ solvents primarily based on their negligible vapour pressure and low flammability [3]. Since Pernak's pioneering study about the ecotoxicity of ILs [4], the environmental impacts of this class of ‘green’ solvents have been gaining attention in academia and industry, particularly with regard to their toxicity and biodegradability [5], [6]. It has emerged that commonly used imidazolium- and pyridinium-based ILs are not as environmentally friendly as previously thought. For instance, such ILs generally showed considerable toxicity to enzymes, microorganisms and cells as well as to whole animals and plants [7]; and most could not be considered as ‘readily biodegradable’ [5].

It has been widely demonstrated that the cations of ILs, especially the head groups, play a major role in toxicity [8], [9]. For example, ILs containing quaternary ammonium and alicyclic cations (morpholium, piperidinium and pyrroliudinium) generally display much lower toxicity than those with aromatic cations such as imidazolium and pyridinium [6]. In addition, introduction of polar hydroxyl, ether and nitrile functional groups into the alkyl side can significantly reduce the toxicity of ILs to acetylcholinesterase (AChE) [8]. Based on current restricted knowledge about the relationships between structures and properties of ILs, a T-SAR (Thinking in terms of Structure-Activity Relationships) strategy has been proposed for rational design of novel ‘greener’ ILs [8]. According to this strategy, cholinium is a promising candidate as the IL cation, since the quaternary ammonium cation incorporating a polar hydroxyl group is expected to have relatively low toxicity. In addition, choline, a biologically widespread molecule that is an essential micronutrient, is able to degrade completely under aerobic conditions [10]. Recently, a variety of cholinium-based ILs has been synthesized [11], [12]. Indeed, these cholinium-based ILs have been reported to have low toxicity [13]–[15], and most examples tested are readily biodegradable [14], [16].

**Figure 1:**
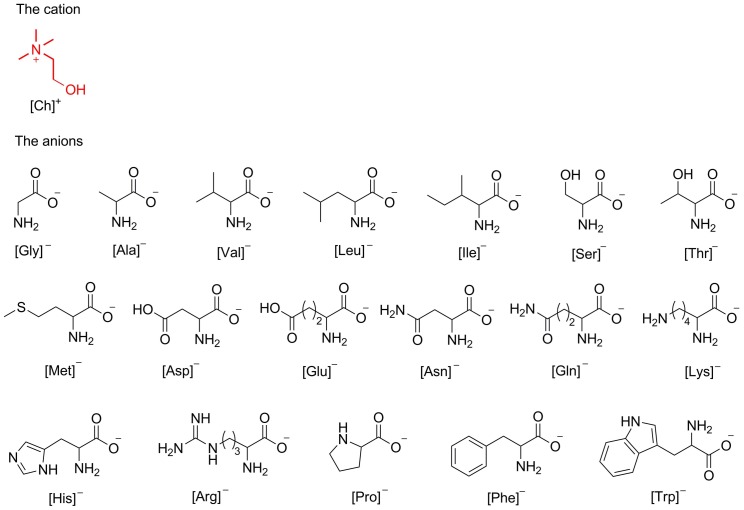
Structures of the cation and anions of [Ch][AA] ILs.

The anion has also proven to contribute to the overall toxicity of ILs [14], [17], although its effect has often been overlooked previously, possibly due to the limited anion types reported. Amino acids, which have prolific structural diversity as the anionic components of ILs, offer the opportunity for detailed structure-based study of the effect of the anion on IL toxicity and other properties, although to our knowledge toxicity and biodegradation studies of amino acid-based ILs have not been reported previously. Amino acids, as one of the most abundant classes of organic compounds in nature, are excellent feedstocks for synthesis of ILs [18]; a number of amino acid-based ILs, in which amino acids act as the cations or anions, have emerged [19], [20]. Recently, our group reported synthesis as well as chemical and physical characterization of 18 novel ILs with cholinium as the cation and amino acids as the anions ([Ch][AA], Figure 1) [21]; additionally, these ILs were found to be highly effective solvents for lignocellulosic biomass pretreatment via selective lignin removal [21], [22], and to be excellent catalysts for organic synthesis [11], [12]. Before application of such ILs on the industrial scale it is important to assess the likely effects of their release into the environment and to obtain a structure-based understanding of environmental fate of this class of ILs in order to permit selection or design of appropriate, less toxic and readily biodegradable ILs. The aim of this work was to evaluate the toxicity of [Ch][AA] ILs to enzymes and representative bacteria (*Escherichia coli*, *Staphylococcus aureus*, *Salmonella enteritidis*, and *Listeria monocytogenes*), and to assess their biodegradability using wastewater microorganisms through two different methods – closed bottle and CO_2_ headspace tests. Consequently, we have been able to evaluate the effect of the nature of the anions on the toxicity and biodegradability of the ILs.

## Materials and Methods

### Biological and chemical materials

[Ch][AA] ILs were synthesized and characterized as described recently by our group [21]. 1-Butyl-3-methylimidazolium tetrafluoroborate ([Bmim][BF_4_]) was bought from Lanzhou Institute of Chemical Physics, China. Cholinium acetate ([Ch][AcO]) was prepared as described recently [21], and its NMR spectra were shown to be the same as reported previously [14]. AChE type VI-S (from the electric eel), acetylthiocholine iodide, 5,5′-dithio-bis-(2-nitrobenzoic acid) (DTNB) were purchased from Sigma–Aldrich, USA. The strains *E. coli* ATCC 8739, *S. aureus* ATCC 29213, *S. enteritidis* ATCC 14128 and *L. monocytogenes* ATCC 19115 were bought from the China Medical Culture Collection. All other chemicals were from commercial sources and were of the highest purity available.

### AChE inhibition assays

A colorimetric assay based on reduction of DTNB was used to measure AChE inhibition as described previously [23], [24], in the presence of a phosphate buffer system in order to eliminate the effects of pH changes on enzyme activity. Briefly, IL solutions were prepared with 0.2 M phosphate buffer (pH 8.0) in 96-well microtiter plates. The enzyme (0.2 U mL^−1^, plus 0.25 mg mL^−1^ bovine serum albumin in the same phosphate buffer), and DTNB (2 mM, plus 0.185 mg mL^−1^ NaHCO_3_ in the same phosphate buffer) were added to each well and kept at 37 °C for 5 min, and then the reaction was initiated by adding acetylthiocholine iodide (2 mM in the same phosphate buffer). In the experimental reactions, the final concentrations of DTNB, acetylthiocholine iodide and AChE were 0.5 mM, 0.5 mM and 0.05 U mL^−1^, respectively. Except where stated otherwise, the concentration range used to calculate EC_50_ of the [Ch][AA] ILs was 0–50 mM. However, for strongly alkaline [Ch][AA] ILs such as [Ch][Lys], the maximum concentration used was 20 mM in order to avoid changes in enzyme activity due to pH changes that would be caused if the IL exceeded the buffering capacity of the phosphate buffer. Acidic amino acid-derived ILs ([Ch][Asp] and [Ch][Glu]) were used up to 80 mM. The control compounds cholinium chloride ([Ch][Cl]), [Bmim][BF_4_] and acetone were used at concentration ranges of 0–80 mM, 0–50 mM and 10 mM–2.5 M, respectively. Control experiments without enzyme were carried out under otherwise identical conditions. Reduction of DTNB was monitored at 405 nm and at 10 second intervals in a microplate reader (Wallac 1420 VICTOR3, PerkinElmer, Singapore) over a period of 5 min. Enzyme activity was expressed as ΔOD min^−1^ obtained via linear regression analysis. All assays were performed at least in triplicate.

The IL concentration that resulted in 50% reduction of the enzyme activity (EC_50_) was calculated from response curves of percent inhibition plotted against the decadic logarithm of the concentration of the IL, by nonlinear fitting using Origin Software (OriginLab Corporation 8.5, USA) with a dose-response curve model. All data were analyzed by One-Way analysis of variance using SPSS 11.5 (SPSS Inc., USA). EC_50_ values are presented as mean ± standard deviation (SD) from at least three repeats, and confidence intervals (*α* = 0.05) of EC_50_ are also presented. The One-Way ANOVA was used to judge if the difference between the different ILs groups was significant. When the *p* value was<0.05, the difference was considered to be significant.

### Antimicrobial activity

Antimicrobial activity was measured via the tube dilution method as described previously [25]. Briefly, *E. coli*, *S. aureus*, *S. enteritidis* and *L. monocytogenes* were cultured in Mueller-Hinton broth for 24 h at 37 °C. A microorganism suspension of 10^6^ cfu mL^−1^ (cfu, colony forming units) was prepared from each culture. IL solutions (31.3–1500 mM) were prepared with sterilized Mueller-Hinton broth, and then were sterilized by filtration (0.45 µm pore-diameter membrane). The various permutations of microorganism suspension (50 µL) and IL solution (50 µL) were mixed in 96-well microtiter plate. Growth of the microorganisms was determined visually after incubation for 24 h at 37 °C. Controls were conducted under the same conditions: negative controls without microorganisms and positive controls without ILs. The lowest concentration at which there was no visible growth (turbidity) was taken as the minimal inhibitory concentration (MIC). Samples of 20 µL from each well were spread onto Mueller-Hinton agar medium with inactivates (0.3% lecithin, 3% polysorbate 80 and 0.1% L-cysteine) and incubated for 48 h at 37 °C. The lowest concentration of the IL that killed 99.9% or more of the test bacterium was taken as the minimum biocidal concentration (MBC).

### Closed bottle test

Closed bottle tests were conducted according to OECD guideline 301 D. The mineral medium was composed of 8.5 mg L^−1^ KH_2_PO_4_, 21.75 mg L^−1^ K_2_HPO_4_, 33.4 mg L^−1^ Na_2_HPO_4_ · 2H_2_O, 0.5 mg L^−1^ NH_4_Cl, 27.5 mg L^−1^ CaCl_2_ · 2H_2_O, 22.5 mg L^−1^ MgSO_4_ · 7H_2_O and 0.25 mg L^−1^ FeCl_3_. Solutions containing the test substances at 3 mg L^−1^ were prepared in aerated mineral media and then inoculated with aerated secondary effluent from Xilang wastewater treatment plant (Guangzhou, China). A control with microorganisms, but without ILs, was conducted in parallel as the oxygen blank. Sodium benzoate was used as the reference substance. Triplicates of test control, reference samples and test ILs were kept in the dark at 25±1 °C. The standard test period was 28 days, and samples were taken for the determination of dissolved oxygen (3-Star, Thermo, USA) at seven day intervals. The biodegradability was calculated by dividing the biochemical oxygen demand (BOD; expressed as mg of O_2_ per mg of the test IL) by the theoretical oxygen demand (ThOD).

## Results and Discussion

### Toxicity toward AChE

AChE is an essential component of the nervous system of nearly all higher organisms including humans [26]. It was used as the test enzyme because its active center is highly conserved and well known, and this enzyme has been widely used for toxicity assay of ILs [8], [24], [27], [28]. The toxicity of the [Ch][AA] ILs toward AChE was expressed as EC_50_ values, which could be calculated from concentration-response curves. Figure S1 (available in File S1) shows the concentration-response curves of representative [Ch][AA] ILs and controls, which comprised the conventional IL [Bmim][BF_4_], [Ch][Cl] and the biodegradable organic solvent acetone. All the [Ch][AA] ILs, in which the charged group of the cation is the quaternary ammonium head group of choline, exhibited much lower toxicity to AChE than the traditional IL [Bmim][BF_4_] (entries 1–18 *vs.* entry 20, Table 1). [Bmim][BF_4_] has been demonstrated to be less harmful toward various enzymes than corresponding IL containing the chloride anion [29]–[31] and hence was used as a relatively low toxicity conventional IL with which to compare the enzyme inhibition due to the [Ch][AA] ILs. The inhibitory effects of these [Ch][AA] ILs were approximately an order of magnitude weaker than that of [Bmim][BF_4_], which is in agreement with previous results where high toxicity of ILs containing imidazolium cations was attributed to the formation of strong π–π interactions between the aromatic imidazolium head group of the cation and the side chain of amino acid Trp 279 present in the peripheral anionic site of AChE [8]. Moreover, with the exception of [Ch][Phe] and [Ch][Trp], all the [Ch][AA] ILs exhibited comparable or even slightly lower toxicity than [Ch][Cl], a naturally occurring micronutrient (entry 19, Table 1). However, all the [Ch][AA] ILs appeared to be considerably more toxic than acetone (entry 21, Table 1). Interestingly, all the [Ch][AA] ILs showed comparable low inhibition toward the enzyme, with EC_50_ values of 2400–3800 µM, which might be attributed to the enzyme-compatible cholinium head group. Nonetheless, there were significant variations in the inhibitory effects of different [Ch][AA] ILs (entries 1–18, Table 1). For example, in several instances the elongation of the amino acid side chain resulted in greater toxicity (entries 1–3, Table 1). Similar effects were also observed when the size of the side chain was increased in amino acids that have the same functional group, as is demonstrated by the following pairs of ILs: [Ch][Ser] and [Ch][Thr] (entries 6 and 7, Table 1); [Ch][Asp] and [Ch][Glu] (entries 9 and 10, Table 1); [Ch][Asn] and [Ch][Gln] (entries 11 and 12, Table 1). These results highlight the role of the lipophilicity of the anion in toxicity. The active site of AChE is located at the bottom of a deep and narrow gorge that is lined with lipophilic aromatic amino acid residues [32]. The data suggest that the hydrophobic interactions between side chains of the amino acid anion components of the ILs and lipophilic amino acid residues in this cavity within the enzyme became stronger with the increase of the chain length of the amino acid component of the IL, thus enhancing the inhibitory effect of the IL on the enzyme. However, among the amino acids with hydrophobic aliphatic side chains, increase in the size of the side chain beyond valine led to lower inhibitory effects (entries 4 and 5 *vs.* entries 1–3, Table 1). As mentioned above, the entrance of the active site is deep and narrow, and therefore the ILs with amino acids with longer side chains might be less potent inhibitors of the enzyme because of steric hindrance that may prevent the amino acid entering the active site of the enzyme. Introduction of hydrophilic groups such as hydroxyl, carboxyl and amino into the amino acid generally decreased the inhibitory potentials of the ILs (entries 6, 7 and 9–15, Table 1). Among all [Ch][AA] ILs tested, [Ch][Trp] and [Ch][Phe] were the two most potent inhibitors of AChE (entries 17 and 18, Table 1). There are approximately 40% aromatic amino acid residues in the gorge of AChE, which may bind readily with the aromatic systems in [Ch][Trp] and [Ch][Phe] via π–π interactions. This might account for the strong inhibitory properties of these two ILs. Compared with [Ch][Phe], the slightly greater inhibition caused by [Ch][Trp] may be attributed to the stronger π-stacking interaction of its larger aromatic system (indole ring) with aromatic amino acid residues in the gorge. Similarly, Arning et al. found that the introduction of aromatic systems into the side chain of the cations led to higher toxicity [8].

**Table 1:** Toxicity of [Ch][AA] ILs toward AChE. a

Entry	ILs	EC_50_±SD / µM	Confidence Interval (95%, α = 0.05)	n b
1	[Ch][Gly]	3830±170	3652−4018	6
2	[Ch][Ala]	3330±100	3168−3489	4
3	[Ch][Val]	3060±120	2874−3244	4
4	[Ch][Leu]	3360±50	3288−3436	4
5	[Ch][Ile]	3570±70	3466−3681	4
6	[Ch][Ser]	3560±150	3321−3804	6
7	[Ch][Thr]	3450±150	3260−3642	5
8	[Ch][Met]	3130±140	2902−3358	4
9	[Ch][Asp]	3810±90	3669−3904	4
10	[Ch][Glu]	3720±70	3612−3821	4
11	[Ch][Asn]	3940±50	3863−4026	4
12	[Ch][Gln]	3510±160	3315−3699	5
13	[Ch][Lys]	3480±130	3286−3683	4
14	[Ch][His]	3630±40	3578−3692	4
15	[Ch][Arg]	3670±70	3552−3778	4
16	[Ch][Pro]	3370±160	3115−3616	4
17	[Ch][Phe]	2740±100	2585−2900	4
18	[Ch][Trp]	2450±20	2413−2489	3
19	[Ch][Cl]	3160±90	3020−3292	4
20	[Bmim][BF_4_]	330±10	315−353	4
21	Acetone	427670±12990	407002−448336	4

a Conditions: DTNB (0.5 mM), AChE (0.05 U mL^−1^), acetylthiocholine iodide (0.5 mM), and ILs (0–80 mM). Enzyme kinetics were recorded at 37 °C at 10 second intervals for 10 min at 405 nm.

b Number of times the experiment was performed.

To further investigate the molecular toxicity of [Ch][AA] ILs, catalase was used as another model enzyme for inhibition studies (Table S1, available in File S1), which has been used previously for evaluation of environmental impact of ILs [33]. The ILs with the various amino acid anions showed a very similar trend of varying toxicity toward catalase compared to that toward AChE, although the concentrations of ILs required to inhibit catalase were 600–800 times greater than those needed to cause the same inhibition of AChE. Since it is a product of the natural AChE reaction, choline in the ILs might inhibit AChE by product inhibition, which would in part account for the fact that the ILs are more toxic against AChE than catalase.

### Toxicity toward bacteria

Antibacterial activities of [Ch][AA] ILs were estimated using four representative bacteria: *E. coli* and *S. enteritidis* (Gram-negative rods); *L. monocytogenes* (Gram-positive rod); and *S. aureus* (Gram-positive coccus). The results are expressed as MIC and MBC (Table 2). It was found that [Ch][AcO] and [Ch][Cl] showed lower toxicity toward the test bacteria than [Ch][AA] ILs. However, like the enzyme inhibitory results above described, the imidazolium-based [Bmim][BF_4_] was much more toxic than these novel ILs. In most instances the Gram-negative bacteria were more susceptible to the ILs than the Gram-positive bacteria tested, which is consistent with a previous study where the most resistant bacterium to a range of imidazolium and pyridinium ILs was found to be the Gram-positive coccus *S. aureus* [34]. However, these results were in contrast to the general trend that Gram-negative bacteria have higher resistance to biocides than Gram-positive bacteria, because of the presence of an extra outer lipopolysaccharide membrane on the cell walls of the former [35]. This implied that a different mechanism might occur in the toxicity of these novel ILs compared to traditional biocides. The [Ch][AA] ILs exhibited several orders of magnitude weaker antimicrobial effects than imidazolium- and pyridinium-based ILs [25], [34], which might be attributed to the low toxicity of the cholinium cation [6]. This was confirmed by the observation that the [Ch][AA] ILs showed similar microbial toxicity to cholinium alkanoate ILs reported previously [14].

**Table 2:** Toxicity of [Ch][AA] ILs toward bacteria. a

		*E. coli*		*S. enteritidis*		*L. monocytogenes*		*S. aureus*	
Entry	ILs	MIC	MBC	MIC	MBC	MIC	MBC	MIC	MBC
1	[Ch][Gly]	125	250	93.8	125	250	500	250	500
2	[Ch][Ala]	46.9	93.8	62.5	93.8	188	250	188	250
3	[Ch][Val]	46.9	62.5	46.9	62.5	93.8	125	93.8	125
4	[Ch][Leu]	46.9	62.5	46.9	62.5	46.9	62.5	93.8	125
5	[Ch][Ile]	46.9	62.5	46.9	62.5	46.9	62.5	93.8	125
6	[Ch][Ser]	31.3	62.5	46.9	62.5	93.8	125	125	250
7	[Ch][Thr]	31.3	62.5	46.9	62.5	93.8	125	125	250
8	[Ch][Met]	31.3	62.5	31.3	62.5	125	250	125	250
9	[Ch][Asp]	500	750	125	188	500	750	750	750
10	[Ch][Glu]	500	750	125	188	500	750	750	750
11	[Ch][Asn]	93.8	125	93.8	125	250	250	188	250
12	[Ch][Gln]	125	188	93.8	125	188	250	125	188
13	[Ch][Lys]	31.3	46.9	31.3	48.7	62.5	93.8	62.5	93.8
14	[Ch][His]	46.9	62.5	46.9	62.5	93.8	125	93.8	125
15	[Ch][Arg]	46.9	93.8	46.9	62.5	93.8	125	93.8	125
16	[Ch][Pro]	46.9	62.5	46.9	62.5	62.5	93.8	93.8	125
17	[Ch][Phe]	31.3	46.9	31.3	46.9	46.9	62.5	62.5	93.8
18	[Ch][Trp]	23.4	31.3	23.4	31.3	31.3	46.9	46.9	62.5
19	[Ch][AcO]	500	750	750	750	500	750	500	750
20	[Ch][Cl]	>750	>750	>750	>750	>750	>750	>750	>750
21	[Bmim][BF_4_]	2.3	4.6	2.3	2.3	2.3	4.6	2.3	4.6

a Expressed as minimal inhibitory concentrations (MIC, mM) and minimum biocidal concentrations (MBC, mM). MIC and MBC concentrations (mM) were obtained from four repeated experiments. For the substance where complete growth inhibition was not observed, values are presented as more than the highest tested concentration (e.g.>750).

The toxicity of the [Ch][AA] ILs increased with elongation of the amino acid side chain (entries 1–3 and 8, Table 2), possibly due to the increase of lipophilicity of the anions. A similar effect was described previously, where there was a strong correlation between the toxicity of the IL and the carbon chain length of alkanoate anions [14]. However, this effect was not apparent when the amino acid anions changed from from valine to leucine or isoleucine (entries 3–5, Table 2), possibly due to the branching of the side chain in these amino acids, which generally reduces lipophilicity. As shown in entries 6, 7 and 9–15 (Table 2), the introduction of polar groups including hydroxyl, carboxyl and amino into the anions presented a range of toxicity effects. Addition of a hydroxyl group to the side chain of the amino acid resulted in a slight increase in antibacterial activity (entry 2 *vs.* entry 6, Table 2). In contrast, the carboxyl group conferred low toxicity on [Ch][Asp] and [Ch][Glu] (entries 9 and 10, Table 2), which are the least toxic among the [Ch][AA] ILs tested. The amide analogs [Ch][Asn] and [Ch][Gln] were slightly more toxic (entries 11 and 12, Table 2). The ILs where the amino acid side chains contained basic functional groups exhibited high toxicity (entries 13–15, Table 2), which was comparable to the toxicity of [Ch][Val]. The great difference in toxicity between [Ch][AA] containing amino acids with acidic and basic side chains might be partly explained by the alkalinity of the ILs (entries 9, 10 *vs.* entries 13–15, Table 2). In 5 mM solution, the pH values of the [Ch][AA] ILs containing basic amino acids anions were much higher (pH 10.0–11.3) than similar solutions of the ILs containing acidic amino acids, which had pH values around 7 [21]. The environmental pH values would significantly increase in the presence of these basic amino acid-based ILs at high concentrations, which may result in greater toxicity to bacteria. Among the ILs tested, [Ch][Phe] and [Ch][Trp] were overall the most toxic to bacterial cells (entries 17 and 18, Table 2), which suggested that the incorporation of aromatic systems into the anions substantially increased the toxicity of the ILs.

### Biodegradability

Aerobic biodegradability of the [Ch][AA] ILs was evaluated by two standard methods - the closed bottle and CO_2_ headspace tests, in which [Ch][AA] ILs were added to aerobic aqueous media inoculated with wastewater microorganisms, and the depletion of dissolved O_2_ or the evolution of CO_2_ was determined periodically. The biodegradability of the [Ch][AA] ILs was compared with [Ch][AcO] and the reference compound sodium benzoate (Tables 3 and S2, available in File S1). The biodegradability was expressed as a percentage of the theoretical maximum. Generally, the biodegradation test methods are considered to be valid if the reference compound of >60% is biodegraded within 14 days. In this study, the biodegradability of sodium benzoate was measured as >75% in 14 days by both methods, thus indicating the validity of the two test methods. According to the Organization for Economic Cooperation and Development (OECD), the compounds which reach a biodegradation level of >60% after 28 days are considered to pass the biodegradation test [5]. Biodegradability of all [Ch][AA] ILs was more than 60% after 28 days by the closed bottle (Table 3) and CO_2_ headspace tests (Table S2 in File S1), so all of these ILs can be referred to as ‘readily biodegradable’. Most of the [Ch][AA] ILs showed greater susceptibility to aerobic biodegradation than [Ch][AcO], which was reported previously to be a low toxicity IL [14]. The high degrees of biodegradation of the ILs could be attributed to the cholinium cation as well as the presence of carboxyl and primary amino groups in the anions [10].

**Table 3:** Biodegradation of ILs determined by the closed bottle test.^a^

		Biodegradability (%)			
Entry	ILs	7 days	14 days	21 days	28 days
1	[Ch][Gly]	58.3±2.3	72.3±0.1	75.0±1.2	82.6±1.1
2	[Ch][Ala]	61.9±0.8	66.8±0.1	77.6±1.2	80.0±0.4
3	[Ch][Val]	49.6±0.1	61.3±0.8	65.6±0.5	69.4±0.6
4	[Ch][Leu]	46.3±0.8	68.5±1.6	70.7±0.9	72.4±0.1
5	[Ch][Ile]	57.3±2.4	68.4±0.1	70.5±0.3	71.6±0.8
6	[Ch][Ser]	53.8±2.2	72.0±0.7	73.5±1.7	80.6±1.5
7	[Ch][Thr]	44.6±2.3	64.9±1.7	70.0±1.1	74.3±1.5
8	[Ch][Met]	54.3±0.3	63.7±0.1	64.7±0.3	66.1±0.9
9	[Ch][Asp]	68.9±1.2	79.5±0.6	86.5±0.5	87.1±0.6
10	[Ch][Glu]	70.0±0.1	71.3±1.1	83.1±1.4	86.3±0.2
11	[Ch][Asn]	53.9±0.5	71.8±1.1	85.7±0.7	87.1±1.2
12	[Ch][Gln]	58.5±2.3	80.5±0.7	83.8±1.5	86.6±1.4
13	[Ch][Lys]	54.4±3.7	62.4±0.4	65.9±1.0	67.7±1.0
14	[Ch][His]	46.5±3.5	60.1±1.2	63.4±0.0	65.3±1.3
15	[Ch][Arg]	59.6±2.3	62.3±0.9	65.3±0.1	67.6±0.2
16	[Ch][Pro]	58.0±0.9	66.4±0.1	68.5±0.9	71.3±0.1
17	[Ch][Phe]	44.0±0.2	68.8±0.2	71.0±1.1	70.8±0.1
18	[Ch][Trp]	55.1±1.1	60.2±0.6	62.7±1.1	65.9±0.2
19	[Ch][AcO]	46.7±0.4	63.6±0.8	66.5±0.5	68.1±1.9
20	Sodium benzoate	60.1±0.2	75.0±1.0	78.5±0.6	81.1±0.8

a Data are shown as means ± SD (n = 3).

Differences in aerobic biodegradability of various [Ch][AA] ILs could be accounted for based on the structures of amino acid anions. The use of amino acids with branched side chain, such as Val, Leu and Ile, as the anions increased resistance to aerobic biological breakdown by microorganisms, leading to the biodegradability of 69–72% (entries 3–5, Table 3) compared to the >80% biodegradation observed with the unbranched side chains of [Ch][Gly] and [Ch][Ala] (entries 1 and 2, Table 3). Similarly, Petkovic et al. reported that ILs with branched chain alkanoate anions were more resistant to fungal attack than their linear isomers [14]. The presence of hydroxyl in the anions led to a slight improvement in biodegradation, which was also been observed in other types of ionic liquids [36]. For instance, due to the replacement of a methyl group with a hydroxyl, the level of biodegradation of [Ch][Thr] was marginally higher than that of [Ch][Val] (74% *vs.* 69%, entry 7 *vs.* entry 3, Table 3). The ILs incorporating carboxylic acid and amide groups showed excellent degradability (>86%, entries 9–12, Table 3). These results are in agreement with Boethling's rules of thumb where the presence of carboxylate groups or hydrolysable bonds such as ester and amide generally increases aerobic biodegradability [10], although they contrast somewhat with the study of Gathergood et al. that reported negligible biodegradation of ILs incorporating an amide group in the cation side chain [37]. The ILs containing basic amino acids were less susceptible to microbial breakdown and degradation levels of 65–68% were obtained (entries 13–15, Table 3). It is unlikely that the poor biodegradation of these ILs stemmed from their strong basicity, because their concentrations were extremely low (3 mg L^−1^) in the test systems. The relatively low biodegradability of [Ch][His] might be attributed to the presence of the heterocyclic imidazole group, which may be unfavorable for biodegradation [10]. Similarly, the biodegradability of [Ch][Trp], containing the heterocyclic indole double ring structure, was slightly lower than that of [Ch][Phe] containing a single aromatic carbon ring structure (71% vs. 66%, entries 16 and 17, Table 3) [10]. It was previously reported that cholinium 2-naphthoxyacetate and cholinium anthracene-9-carboxylate, which contain polycyclic aromatic hydrocarbon ring moieties, failed the biodegradability test [16].

A similar trend that the biodegradability of ILs depends on the structures of amino acid anions was observed in the CO_2_ headspace test (Table S2 in File S1). For example, the ILs containing carboxyl and amide groups showed excellent biodegradability (81–85%, entries 9–12, Table S2 in File S1). In the cases of the ILs with basic amino acid anions, moderate biodegradation (63–66%) was observed (entries 13–15, Table S2 in File S1). Likewise, [Ch][Trp] appeared to be relatively recalcitrant to microbial attack, affording a biodegradation level of 62%.

**Figure 2:**
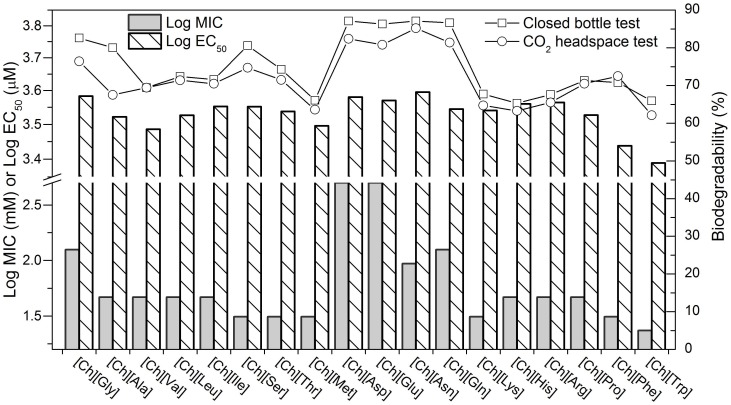
The correlation between toxicity toward AChE (log EC_50_) and *E. coli* (log MIC) and biodegradability.

In the rational design of environmentally benign compounds, a conflict has sometimes been observed between minimizing toxicity and maximizing biodegradability [10]. For example, the elongation of side chain that resulted in enhanced biodegradation of pyridinium-based ILs correlated with an increase in their toxicity [38]. To analyze the correlation between the two properties of [Ch][AA] ILs, their toxicity toward AChE and *E. coli* was compared with the biodegradability data, as depicted in Figure 2. Interestingly, such a conflict between the toxicity and biodegradability did not occur in these [Ch][AA] ILs. With relatively few exceptions, the [Ch][AA] ILs with lower toxicity had higher degradability. For example, the readily biodegradable [Ch][Asp] and [Ch][Glu] displayed low toxicity; and [Ch][Trp] with higher toxicity was found to be more recalcitrant to microbial degradation.

## Conclusions

The [Ch][AA] ILs were found generally to have low toxicity and to be readily biodegradable. In addition, the results presented here reinforce the advantages of use of renewable biomaterials as starting materials for the synthesis of environmentally friendly ILs. The low toxicity and low environmental persistence, combined with their previously demonstrated excellent solvent properties for selective extraction of lignin, indicate that ILs of this type are highly suitable candidates for large-scale applications. More detailed ecotoxicological tests with organisms of different trophic levels including fungi, crustaceans, algae, plants, mammalian cell lines and animals are now necessary to determine whether the [Ch][AA] ILs can be given the accolade of truly ‘green’ chemicals. Also, it will be interesting to apply analytical tools such as HPLC-MS and NMR to trace the biodegradation pathways of the cation and anions in these promising new ILs.

## Supporting Information

File S1 **Supplementary material.**
(DOC)
