# Supplementary material for: Evaluation of Toxicity and Biodegradability of Cholinium Amino Acids Ionic Liquids
Source: PLoS One. 2013 Mar 15;8(3):e59145. doi: 10.1371/journal.pone.0059145 (PMC3598705; doi:10.1371/journal.pone.0059145)
Supplement: File S1 — Supplementary material. (DOC) [file pone.0059145.s001.doc]

**Supplementary material for**

**Evaluation of toxicity and biodegradability of cholinium amino acids ionic liquids**

Xue-Dan Hou,^1,2^ Qiu-Ping Liu,^2^ Thomas J. Smith,^3^ Ning Li,^*1^ Min-Hua Zong^*1^

^1^ State Key Laboratory of Pulp and Paper Engineering, College of Light Industry and Food Sciences, South China University of Technology, Guangzhou, China

^2^ College of Bioscience and Bioengineering, South China University of Technology, Guangzhou, China

^3^Biomedical Research Centre, Sheffield Hallam University, Sheffield, UK S1 1WB

^*^Corresponding authors.

Dr. N. Li, Tel/Fax: +86 20 2223 6669; Email: lining@scut.edu.cn.

Prof. M. H. Zong, Tel: +86 20 8711 1452; Fax: +86 20 2223 6669; Email: btmhzong@scut.edu.cn.

**
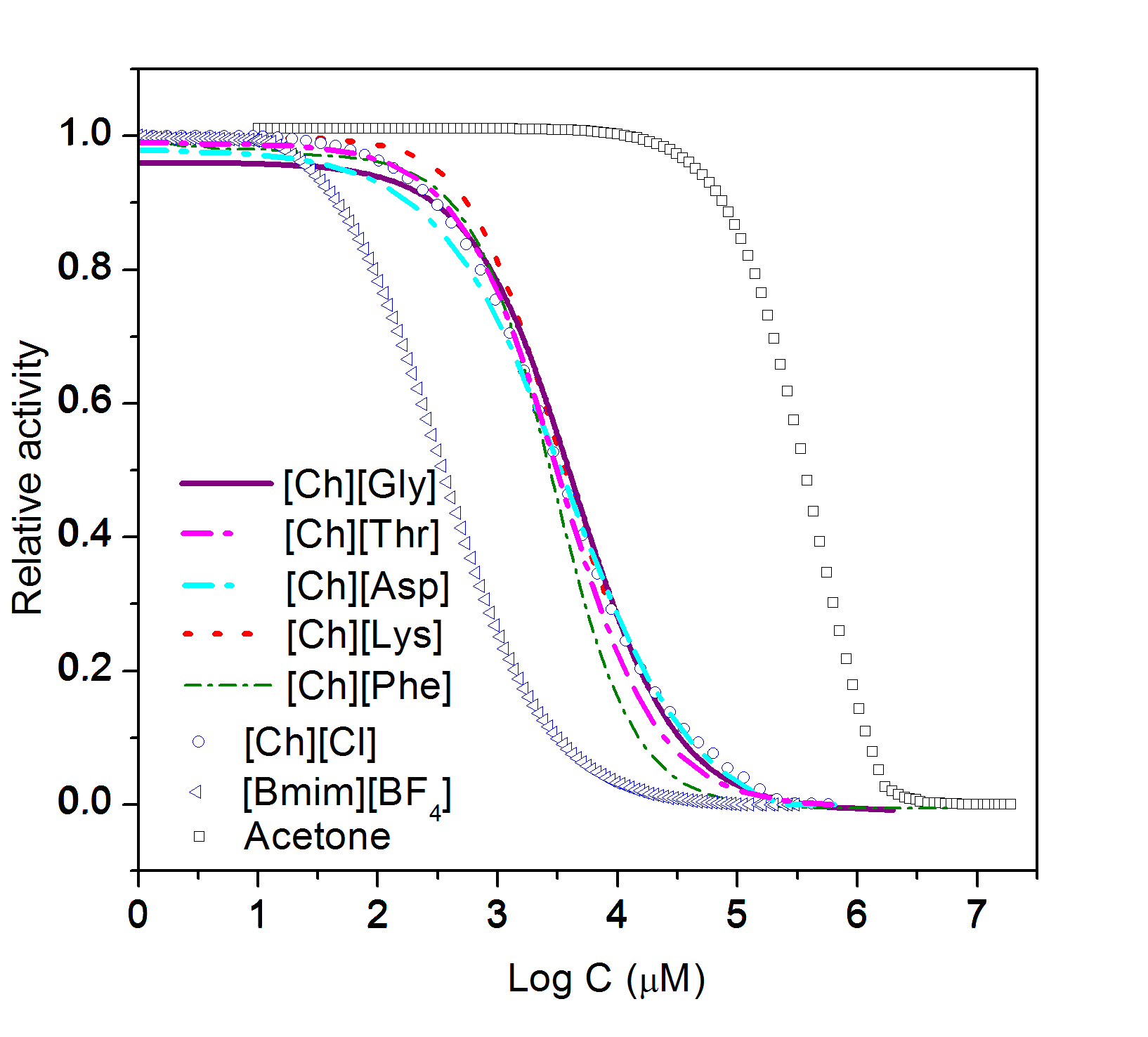
**

**Figure S1 Concentration-response curves of some typical [Ch][AA] ILs and the controls**

Catalase inhibition assay

Catalase activity was determined using a colorimetric assay based on the yellow complex with molybdate and H_2_O_2_, which was described in detail by Goth . The inhibition of catalase was studied as follows. Briefly, 50 μL reaction mixture containing 50 mM H_2_O_2_ and various concentrations of ILs in phosphate buffer (0.2 M, pH 7.4) was prepared in a 96-well microtiter plate, and incubated at 37 ^○^C for 5 min; then 50 μL catalase solution (50 μg mL^-1^ in 0.2 M, pH 7.4 phosphate buffer) was added and incubated at 37 ^○^C for 5 min. The enzymatic reaction was stopped with 100 μL ammonium molybdate (64.8 mM), and the absorbance at 405 nm was measured in a microplate reader (SpectraMax M5, Molecular Devices, USA).

**Table S1. Toxicity of [Ch][AA] ILs toward catalase, expressed as EC_50_ values**

| Entry | ILs | Catalase (EC_50_/M) |
| --- | --- | --- |
| 1 | [Ch][Gly] | 2.30 ± 0.01 |
| 2 | [Ch][Ala] | 2.30 ± 0.01 |
| 3 | [Ch][Val] | 2.12 ± 0.01 |
| 4 | [Ch][Leu] | 2.25 ± 0.01 |
| 5 | [Ch][Ile] | 2.26 ± 0.01 |
| 6 | [Ch][Ser] | 2.29 ± 0.01 |
| 7 | [Ch][Thr] | 2.28 ± 0.01 |
| 8 | [Ch][Met] | 2.21 ± 0.01 |
| 9 | [Ch][Asp] | 2.40 ± 0.01 |
| 10 | [Ch][Glu] | 2.39 ± 0.01 |
| 11 | [Ch][Asn] | 2.41 ± 0.01 |
| 12 | [Ch][Gln] | 2.28 ± 0.01 |
| 13 | [Ch][Lys] | 2.28 ± 0.01 |
| 14 | [Ch][His] | 2.29 ± 0.01 |
| 15 | [Ch][Arg] | 2.30 ± 0.01 |
| 16 | [Ch][Pro] | 2.26 ± 0.01 |
| 17 | [Ch][Phe] | 2.01 ± 0.01 |
| 18 | [Ch][Trp] | 1.95 ± 0.01 |

Conditions: 25 μg mL^-1^ catalase, ILs and 25 mM H_2_O_2_, at 37 ^○^C. After 5 min reaction, the reaction was terminated by adding 100 µL ammonium molybdate (64.8 mM), and the absorbance at 405 nm were recorded. Each experiment was performed in triplicate and values are shown in the form mean ± SD.

CO_2_ headspace test

The CO_2_ headspace test (ISO 14593) was used as one of the tests to evaluate the biodegradability of the ILs . The mineral medium composition is the same as described in the Materials and Methods section of the main text, except that the concentrations of the following components of the medium were increased as indicated: 85 mg L^-1^ KH_2_PO_4_, 217.5 mg L^-1^ K_2_HPO_4_, 334 mg L^-1^ Na_2_HPO_4_·2H_2_O and 5 mg L^-1^ NH_4_Cl. The ILs, as the sole source of carbon and energy, were added at a concentration of 20 mg C L^-1^ to aerated mineral media, and inoculated with aerated secondary effluent collected from Xilang wastewater treatment plant; then, the mixtures were incubated in the dark in sealed vessels with a headspace of air at 25 ± 1 ^○^C and 150 rpm. Biodegradation (mineralization into CO_2_) was determined by measuring the net increase in total inorganic carbon (TIC) levels in the test after subtraction of that in the blank vessels with microorganisms but without the ILs. Sodium benzoate was used as the reference substance, and all the tests ran for 28 days. Three vessels from each group of samples were selected at 7-day intervals, and 3 mL NaOH solution (7 M) was added for total organic carbon determination (LiquiTOC, Elementar, Germany). The biodegradability was expressed as a percentage of the theoretical amount of inorganic carbon (ThIC) that could be produced by complete mineralization of the amount of test compound added initially.

**Table S2.** **Biodegradation of ILs determined via the CO_2_ headspace test.**

|  |  | Biodegradability (%) | | | |
| --- | --- | --- | --- | --- | --- |
| Entry | ILs | 7 days | 14 days | 21 days | 28 days |
| 1 | [Ch][Gly] | 57.8 ± 0.3 | 66.3 ± 0.6 | 74.6 ± 0.4 | 76.4 ± 1.1 |
| 2 | [Ch][Ala] | 62.0 ± 0.6 | 70.5 ± 0.3 | 74.2 ± 0.7 | 76.2 ± 0.8 |
| 3 | [Ch][Val] | 46.4 ± 0.4 | 59.4 ± 0.8 | 64.1 ± 0.3 | 67.5 ± 0.6 |
| 4 | [Ch][Leu] | 52.3 ± 3.1 | 68.6 ± 1.7 | 71.6 ± 1.0 | 71.4 ± 1.0 |
| 5 | [Ch][Ile] | 52.2 ± 0.3 | 66.1 ± 0.3 | 68.3 ± 0.5 | 70.5 ± 0.3 |
| 6 | [Ch][Ser] | 52.5 ± 0.1 | 65.8 ± 0.1 | 73.3 ± 1.1 | 74.7 ± 0.3 |
| 7 | [Ch][Thr] | 36.0 ± 0.4 | 63.7 ± 1.1 | 69.5 ± 0.5 | 71.5 ± 0.8 |
| 8 | [Ch][Met] | 47.8 ± 0.3 | 56.6 ± 0.9 | 62.7 ± 1.1 | 63.6 ± 1.2 |
| 9 | [Ch][Asp] | 65.8 ± 0.1 | 78.9 ± 0.3 | 80.5 ± 0.0 | 82.4 ± 0.7 |
| 10 | [Ch][Glu] | 62.2 ± 0.4 | 71.9 ± 1.1 | 78.0 ± 0.3 | 80.8 ± 0.5 |
| 11 | [Ch][Asn] | 67.2 ± 0.3 | 80.0 ± 0.3 | 83.3 ± 0.6 | 85.2 ± 0.1 |
| 12 | [Ch][Gln] | 57.0 ± 0.6 | 78.0 ± 0.6 | 78.3 ± 0.6 | 81.4 ± 0.1 |
| 13 | [Ch][Lys] | 52.2 ± 0.2 | 61.6 ± 1.2 | 63.1 ± 0.4 | 64.7 ± 0.5 |
| 14 | [Ch][His] | 51.1 ± 1.2 | 60.5 ± 0.3 | 61.1 ± 0.5 | 63.3 ± 1.2 |
| 15 | [Ch][Arg] | 53.0 ± 0.5 | 61.7 ± 1.0 | 63.6 ± 0.8 | 65.5 ± 1.2 |
| 16 | [Ch][Pro] | 50.5 ± 2.1 | 65.5 ± 0.3 | 67.2 ± 6.7 | 70.5 ± 3.0 |
| 17 | [Ch][Phe] | 42.0 ± 0.5 | 60.1 ± 0.3 | 71.4 ± 1.1 | 72.5 ± 0.3 |
| 18 | [Ch][Trp] | 53.0 ± 0.5 | 60.7 ± 1.5 | 61.3 ± 0.3 | 62.1 ± 0.3 |
| 19 | [Ch][AcO] | 47.2 ± 0.3 | 60.4 ± 0.2 | 63.3 ± 0.5 | 65.3 ± 0.1 |
| 20 | Sodium benzoate | 57.0 ± 3.5 | 80.8 ± 0.1 | 83.3 ± 0.6 | 84.1 ± 1.3 |

Data are shown as means ± SD.

**References**

Goth L (1991) A simple method for determination of serum catalase activity and revision of reference range. Clinica Chimica Acta 196: 143-151.

Ford L, Harjani JR, Atefi F, Garcia MT, Singer RD, et al. (2010) Further studies on the biodegradation of ionic liquids. Green Chem 12: 1783-1789.
